# Cranial Juvenile Psammomatoid Ossifying Fibroma: A Rare Entity

**DOI:** 10.7759/cureus.42122

**Published:** 2023-07-19

**Authors:** Muhammad Tahir, Zan Ahmed, Osama Elkadi

**Affiliations:** 1 Pathology and Laboratory Medicine, University of South Alabama Health University Hospital, Mobile, USA

**Keywords:** juvenile ossifying fibroma, fibroma, ossifying fibroma, cemento ossifying fibroma, juvenile psammomatoid ossifying fibroma

## Abstract

Juvenile ossifying fibroma (JOF) is an unusual fibro-osseous lesion primarily occurring in children and young adolescents. Anatomically, this lesion could predominantly arise from the bilateral orbits, paranasal sinuses, maxilla, or mandible. Although it is a benign lesion of osseous origin, it is an aggressive variant of ossifying fibroma of the jaw. Due to the aggressive nature of this lesion and its high tendency for recurrence, early radiological detection and prompt surgical treatment are required. The histologic diagnosis of this entity is purely based on hematoxylin and eosin (H&E), but immunohistochemistry and molecular diagnostic studies can also be performed in challenging cases. A thorough histopathological examination of this lesion is recommended because it can easily be mistaken for another benign fibrosis lesion arising at the same anatomical location. Here, we report the case of a juvenile psammomatoid ossifying fibroma (JPOF) occurring in a 12-year-old boy. The tumor is arising at an extracranial location behind the left anterior cranial fossa.

## Introduction

Juvenile ossifying fibroma (JOF) is a rare benign fibro-osseous neoplasm that is most prevalent in children and young adults. The sinonasal, orbital, and jaw bones are the most frequent sites involved by JOF. Cranial JOF is an exceedingly rare presentation, and the World Health Organization (WHO) classifies JOF into two main subtypes: juvenile trabecular ossifying fibroma (JTOF) and juvenile psammomatoid ossifying fibroma (JPOF) [1]. The JTOF subtype is distinguished by its characteristic pattern of fibrillary osteoid and woven bone organized in a trabecular network. The psammomatoid subtype is characterized by small, multiple, uniform ossicles (psammomatoid bodies) embedded in a cellular stroma composed of stellate and spindled cells. Complete surgical resection is often recommended due to the potential for locally aggressive, destructive growth and the risk of local-regional recurrence [2,3]. We present a case of a 12-year-old boy with appreciable proptosis secondary to a large cranial JOF involving the fronto-orbital bone.

## Case presentation

**Figure 1:**
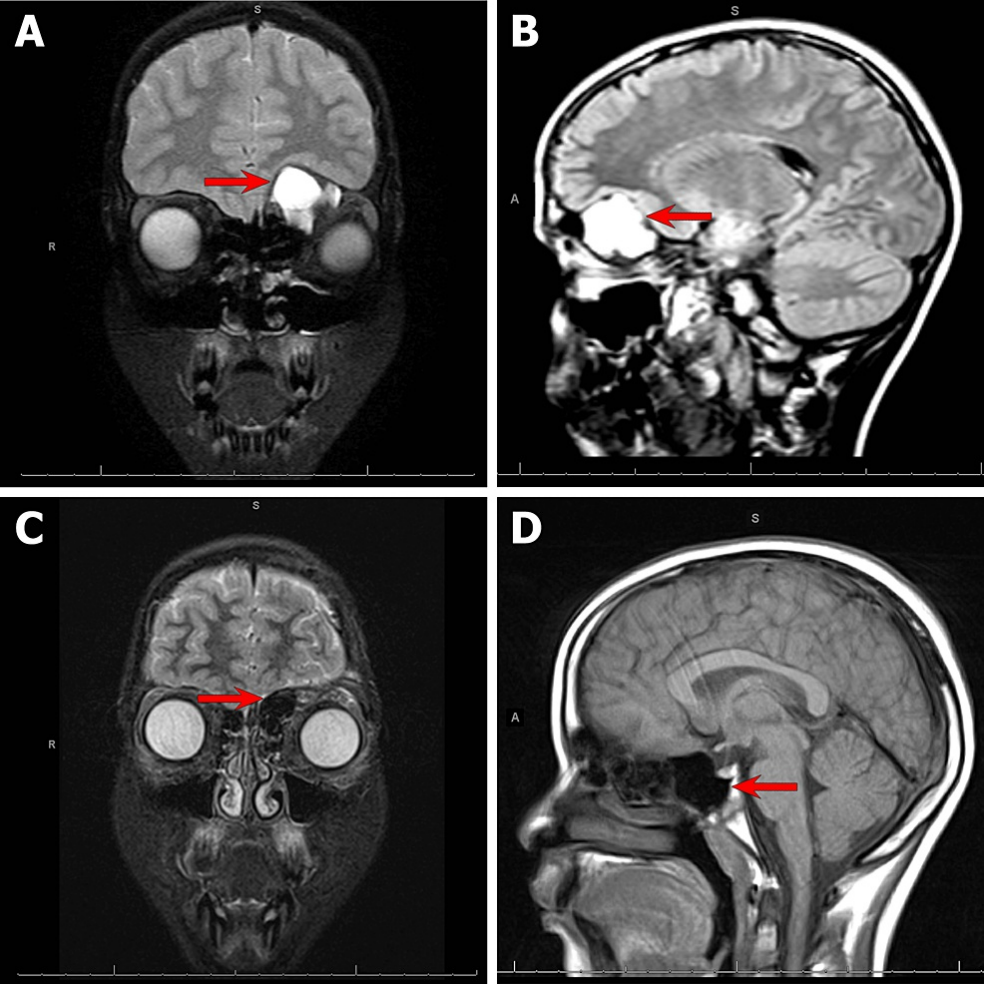
MRI of the head and brain, coronal and sagittal views showing the left extracranial lesion (red arrows in Figures A and B). MRI of the head and brain in coronal and sagittal views after resection (red arrows in Figures C and D) MRI: magnetic resonance imaging

A 12-year-old boy presented to the University of South Alabama Health University Hospital to establish care following a referral from an ophthalmologist. Upon clinical examination, the child has a visual acuity of 20/20 with no peripheral field deficits and prominent proptosis. Magnetic resonance imaging (MRI) revealed an extra-axial left frontal mass expanding the floor of the left anterior cranial fossa, measuring 2.6 x 3.1 x 2.5 cm in longitudinal, transverse, and anteroposterior (AP) dimensions, respectively (Figures 1A, 1B). The lesion shows immediate T1 and elevated T2 signals and is coursing the ventral aspect of the left frontal caudally, encroaching on the left orbit, resulting in proptosis. The lesion imparts irregular peripheral enhancement postcontrast. Surgical intervention was advised to prevent further complications and loss of vision. Resection of the mass and possible orbital reconstruction with tissue grafting were planned and discussed with the patient and family. Surgical resection was done at the University of South Alabama Health University Hospital, and the specimen was sent to the department of pathology for histopathological examination. The patient tolerated the surgical intervention and made an excellent recovery without any complications. Postoperative MRI scans were done to assess for complete resection of the lesion, as shown in Figures 1C, 1D.

**Figure 2:**
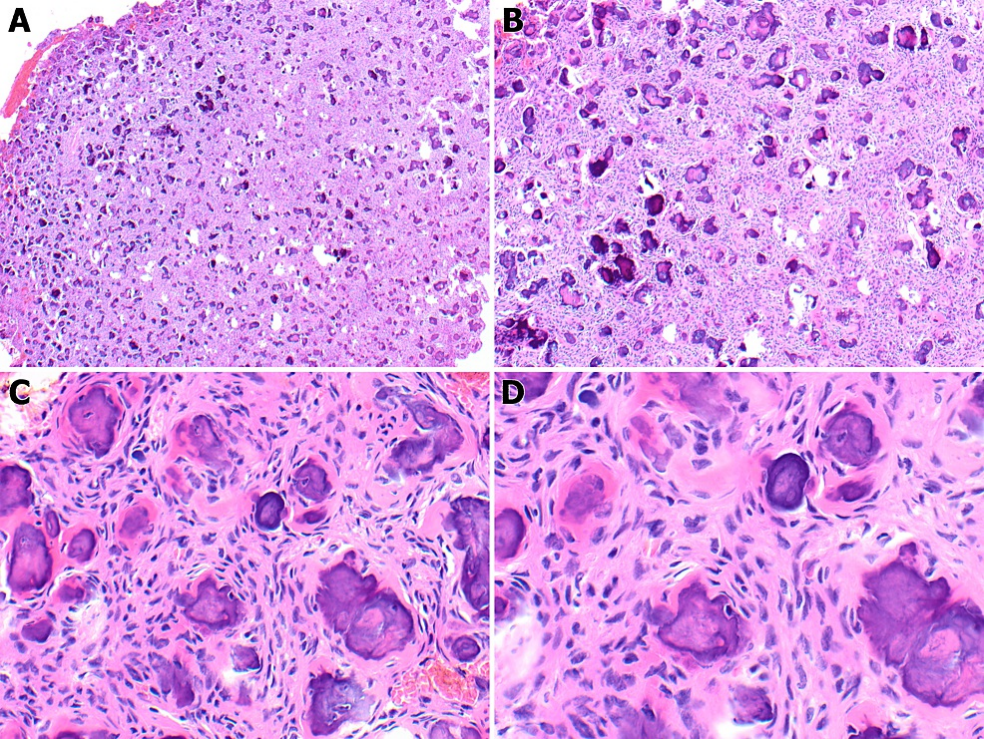
Benign fibro-osseous proliferation (Figure A: 4x, Figure B: 10x). Fibroblastic stroma containing round mineralized ossified microscopic concretions resembling psammoma bodies (Figure C: 40x, Figure D: 60x).

Histopathological examination of the resected tumor shows fragments of cellular connective tissue with spherical ossicles composed of a basophilic center with an eosinophilic rim (Figures 2A, 2B). The tumor is composed of two main components: the matrix and stroma. The matrix consists of calcified structures that are composed of variable amounts of osteoid or woven and lamellar bone or cement-like tissue resembling cementicles or psammoma bodies. The stromal component is composed of plump fibroblastic cells with hyperchromatic nuclei and exceptionally low mitotic activity (Figures 2C, 2D). The ratio between bone and cement-like tissue is variable, with the dominance of one or the other type of calcified tissue.

**Figure 3:**
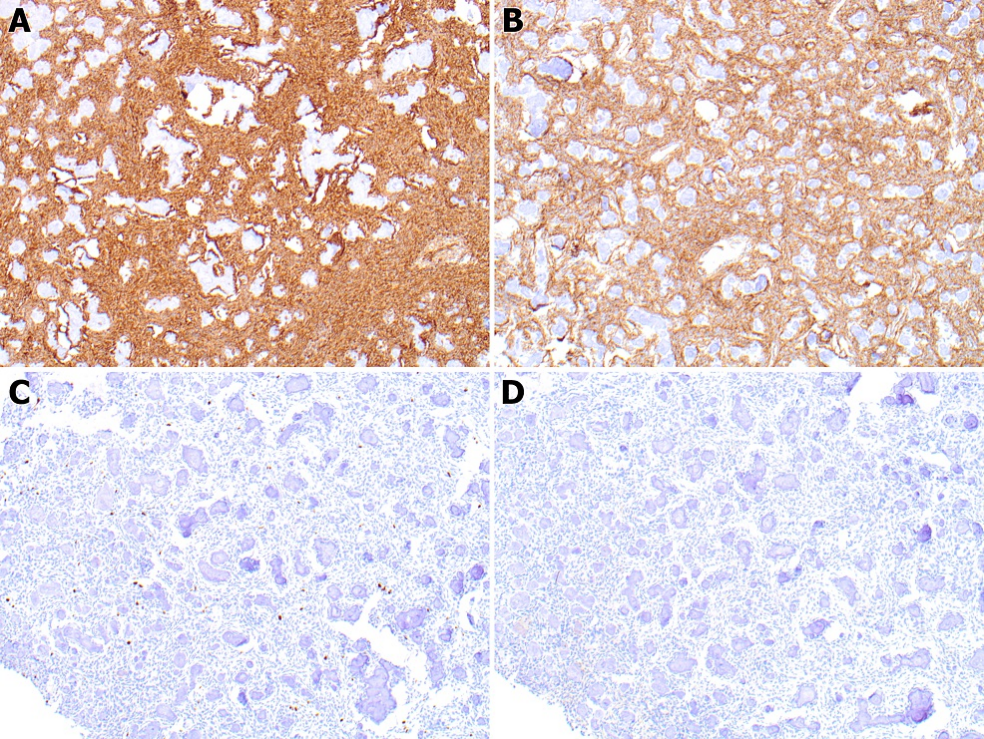
Vimentin is diffusely positive in the stromal component (Figure A: 10x). Normal expression of cytoplasmic beta-catenin (Figure B: 10x) . Low Ki-67 proliferation index (Figure C: 10x). Negative staining for EMA (Figure D: 10x). EMA: epithelial membrane antigen

Immunohistochemistry against antibodies to epithelial Membrane Antigen (EMA), S100, vimentin, glial fibrillary acid protein (GFAP), beta-catenin, and Ki-67 was performed on representative sections of the tumor. The tumor cells are positive for vimentin. Beta-catenin was negative for nuclear translocation. The Ki-67 proliferation index was low (less than 5%) (Figure 3). These results support the diagnostic interpretation, and the diagnosis of JPOF was rendered.

## Discussion

Ossifying fibromas (OF) are the most common fibro-osseous lesions of the orbit and sinonasal tract. The two most common variants of OF are JPOF and JTOF. JPOF has been distinguished because of its characteristic histological features, clinical behavior, and location. It usually occurs in the paranasal sinuses and orbit, accounting for more than 72% of reported cases in the literature, followed by the calvarium (11%), maxilla (10%), and mandible (7%), affecting the young population before age 15 [1,4]. In our case, the most pronounced clinical symptom was proptosis, but patients can present with a variety of symptoms, including optic atrophy, impaired vision, facial swelling, nasal obstruction, headache, peri-orbital pain, bulbar displacement, and sinusitis.

MRI is the best radiological diagnostic tool, and JPOF usually appears well delineated at the periphery with varying degrees of radiopacity and radiolucency. Aggressive lesions may show infiltrative borders with cortical thinning, cystic or solid areas, perforation, hemorrhage, and necrosis [5].

Microscopically, JPOF is composed of fibrous stroma and variable amounts of small to medium-sized round acellular cementicles resembling psammoma bodies embedded within the neoplasm. Morphologically, the cementicles have basaloid centers and an eosinophilic rim around the periphery. Ultrastructural studies found that psammoma bodies possess a dark rim of crystals from which small spicules and needle-like crystalloids project toward the periphery [6]. The stromal component is an integral part of the tumor and can be cellular, which may undergo cystic change, hemorrhage, or coalesce multinucleated giant cells. Mitotic activity is extremely low, but, in some cases, rare mitoses may be seen [7].

Complete surgical excision with negative margins is curative for localized lesions. Radiotherapy is contraindicated because of malignant transformations and late-life complications. The reported recurrence rate of JOPF is about 30-56% [8]. The recurrence is thought to be due to incomplete excision rather than the intrinsic biological properties of the tumor [1,2].

JOF may mimic other bone lesions, such as fibrous dysplasia, cemento-OF, aneurysmal bone cyst, osteogenic sarcoma, or osteoblastoma [8]. It is differentiated from fibrous dysplasia by its relatively well-delineated border from the surrounding tissue [8,9]. Aggressive lesions with marked destruction of adjacent structures mimic osteosarcoma radiographically; however, the lack of periosteal reaction in JOF helps in differentiation [10].

## Conclusions

We present this case to share the histopathological diagnostic features of JPOF and discuss histopathological differential diagnosis with fellow pathologists. Radiographic, clinical, histopathologic, and immunohistochemical features were studied to render the diagnosis of JPOF and to differentiate it from other fibro-osseous lesions, including fibrous dysplasia and other odontogenic tumors. In this case, the treatment of choice, i.e., "radical surgical excision" with negative margins, was adopted. Due to the potential for aggressive behavior, long-term, regular follow-up is recommended.
